# On the uncertainty principle of neural networks

**DOI:** 10.1016/j.isci.2025.112197

**Published:** 2025-03-10

**Authors:** Jun-Jie Zhang, Dong-Xiao Zhang, Jian-Nan Chen, Long-Gang Pang, Deyu Meng

**Affiliations:** 1 Northwest Institute of Nuclear Technology, Xi’an, Shaanxi 710024, China; 2 Key Laboratory of Quark & Lepton Physics of Ministry of Education, Central China Normal University, Wuhan, Hubei 430079, China; 3 School of Mathematics and Statistics and Ministry of Education Key Lab of Intelligent Networks and Network Security, Xi’an Jiaotong University, Xi’an, Shaanxi 710049, China; 4 Macao Institute of Systems Engineering, Macau University of Science and Technology, Macao 999078, China; 5 Pengcheng Laboratory, Shenzhen 518055, China

**Keywords:** Physics, Neural networks, Computer science

## Abstract

In this study, we explore the inherent trade-off between accuracy and robustness in neural networks, drawing an analogy to the uncertainty principle in quantum mechanics. We propose that neural networks are subject to an uncertainty relation, which manifests as a fundamental limitation in their ability to simultaneously achieve high accuracy and robustness against adversarial attacks. Through mathematical proofs and empirical evidence, we demonstrate that this trade-off is a natural consequence of the sharp boundaries formed between different class concepts during training. Our findings reveal that the complementarity principle, a cornerstone of quantum physics, applies to neural networks, imposing fundamental limits on their capabilities in simultaneous learning of conjugate features. Meanwhile, our work suggests that achieving human-level intelligence through a single-network architecture or massive datasets alone may be inherently limited. Our work provides new insights into the theoretical foundations of neural network vulnerability and opens up avenues for designing more robust neural network architectures.

## Highlights

• Neural networks obey an uncertainty principle analogous to quantum mechanics
• Accuracy-robustness trade-off emerges from sharp inter-class boundaries
• Complementarity principle governs conjugate feature learning in AI systems
• Single-network human-level intelligence may face fundamental limitations

## Graphical abstract

**Figure:**
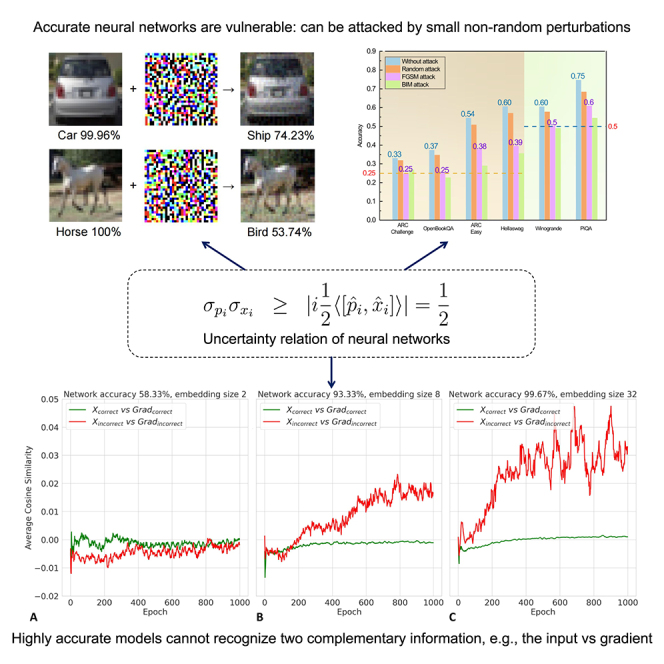

## Introduction

Artificial intelligence (AI), particularly deep neural networks, has revolutionized a wide range of applications, for instance, video generation,^1^ language models,^2^ protein folding,^3^ quantum system analysis,^4^ healthcare,^5^ and autonomous driving.^6^ These advancements highlight the transformative potential of AI across diverse domains, pushing the boundaries of what machines can achieve in both scientific research and real-world applications.

**Figure 1:**
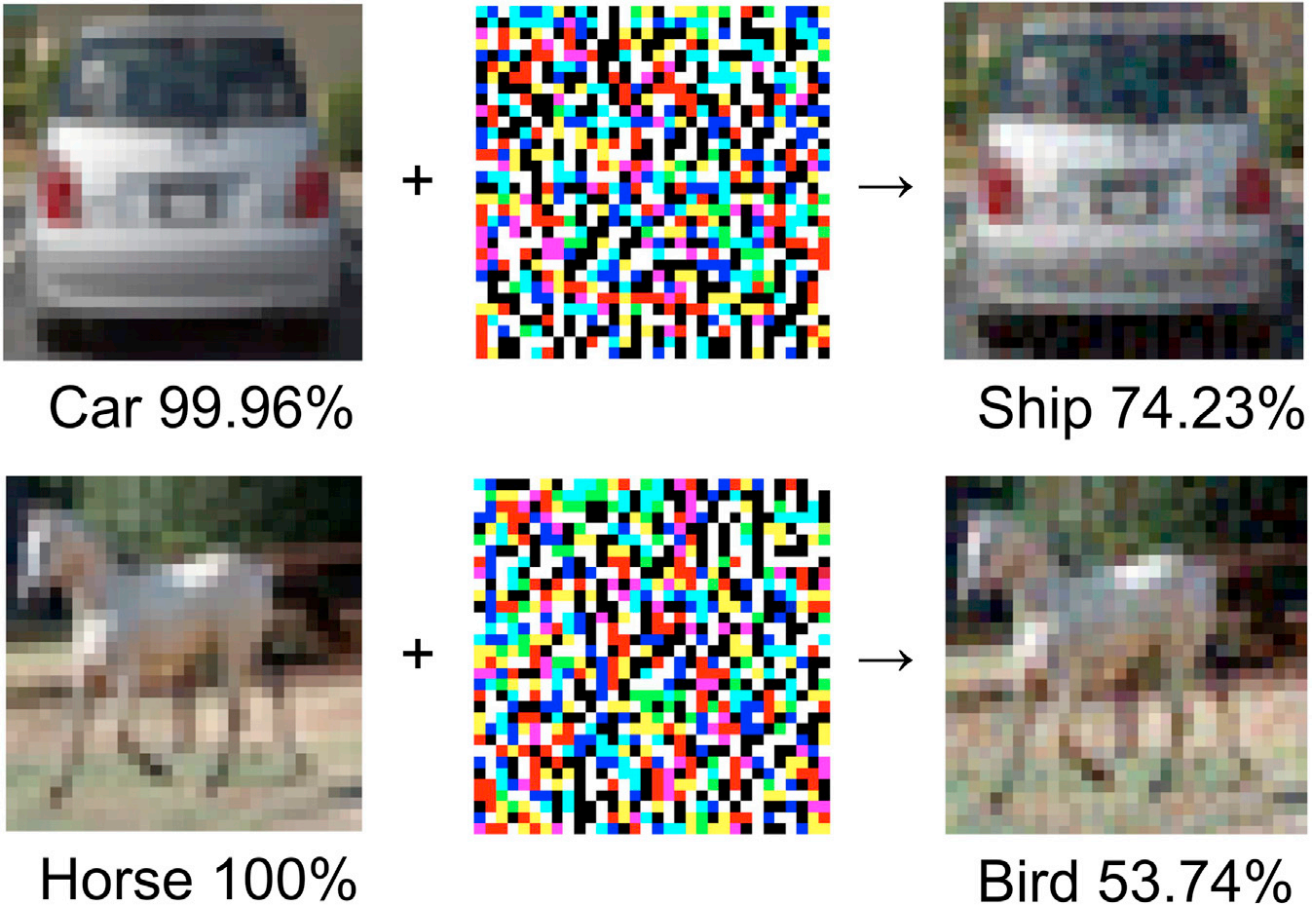
Adding an imperceptible non-random noise to the images, the network will fail to predict the correct label The trained network on CIFAR-10 (an open source dataset containing 10 different types of color images) gives an 89.37% accuracy on the test set and only 9.39% accuracy on the slightly attacked images (category confidence is labeled below each image). The attack, called FGSM (Fast Gradient Sign Method),^16^ is achieved via the transformation $X=X_{0}+\varepsilon \cdot \text{sign}\left(\nabla_{X}l\left(f\left(X,\theta \right),Y^{*}\right){|}_{X=X_{0}}\right)$, where $X_{0}$ denotes the input images of the training set, and $Y^{*}$ is the true label for image $X_{0}$. *X* denotes the image to be attacked, ε=8/255, and $\text{sign}\left(\nabla_{X}l\left(f\left(X,\theta \right),Y^{*}\right){|}_{X=X_{0}}\right)$ gives the non-random noise with $l\left(f\left(X,\theta \right),Y^{*}\right)$ denoting the loss function. *θ* denotes the weights of the trained network.

However, despite their remarkable success, a growing body of research has revealed a critical vulnerability: well-designed and trained AI models exhibit significant fragility when confronted with subtle, non-random perturbations.^7^^,^^8^^,^^9^^,^^10^^,^^11^^,^^12^^,^^13^^,^^14^^,^^15^ These perturbations, known as adversarial attacks (Figure 1), involve adding imperceptible noise to input data, which can drastically degrade the performance of a neural network.

Compared to classical models, the vulnerability of deep neural networks exhibits several distinctive characteristics: the impact of adversarial perturbations is both unpredictable and profound, often leading to catastrophic failures in model performance^8^^,^^9^; moreover, higher accuracy networks tend to be more susceptible to such attacks^10^^,^^11^; efforts to enhance robustness, such as adversarial training where perturbed data are incorporated into the training set,^17^^,^^18^^,^^19^ often result in a trade-off, with improved robustness coming at the cost of reduced accuracy. These interconnected observations suggest an inherent tension between accuracy and robustness in neural networks, indicating a fundamental limitation rooted in these models.

Classical approximation theorems assert that neural networks can approximate continuous functions with arbitrary precision,^20^^,^^21^^,^^22^^,^^23^ implying that stable functions should, in theory, yield stable solutions. Yet, the persistent accuracy-robustness trade-off challenges this assumption. If this trade-off arises solely from network architecture or data acquisition, it might be mitigated through improved engineering. However, if it is intrinsic to the foundational principles of deep learning, a deeper theoretical understanding is required. To date, no definitive explanation for this phenomenon has been established.

In this study, we propose a theoretical framework that attributes the accuracy-robustness trade-off to an uncertainty principle analogous to that in quantum mechanics, which posits that certain pairs of properties cannot be simultaneously determined with arbitrary precision. Translating this concept to neural networks, we argue that a network cannot simultaneously extract two complementary features with maximal accuracy. We substantiate this framework with both analytical and experimental evidence, demonstrating that this uncertainty principle is a universal characteristic of neural networks.

Our findings reveal that the complementarity principle,^24^ a cornerstone of quantum physics, applies to neural networks, imposing fundamental limits on their capabilities in simultaneous learning of conjugate features. Meanwhile, our work suggests that achieving human-level intelligence through a single-network architecture or massive datasets alone may be inherently limited.

## Results

### Neural networks are vulnerable under small imperceptible attacks

As illustrated in Figure 1, trained neural networks can fail under non-random attacks. These phenomena, typically constructed using the gradient of the loss function with respect to the inputs, differ significantly from classical algorithms, which are robust to small input variations. Furthermore, the vulnerability of these neural networks is related to their accuracy—more accurate neural networks tend to be more sensitive to gradient-based attacks, indicating a trade-off between accuracy and robustness.^13^^,^^16^^,^^19^^,^^25^^,^^26^^,^^27^^,^^28^

### Phenomena in image classification

**Figure 2:**
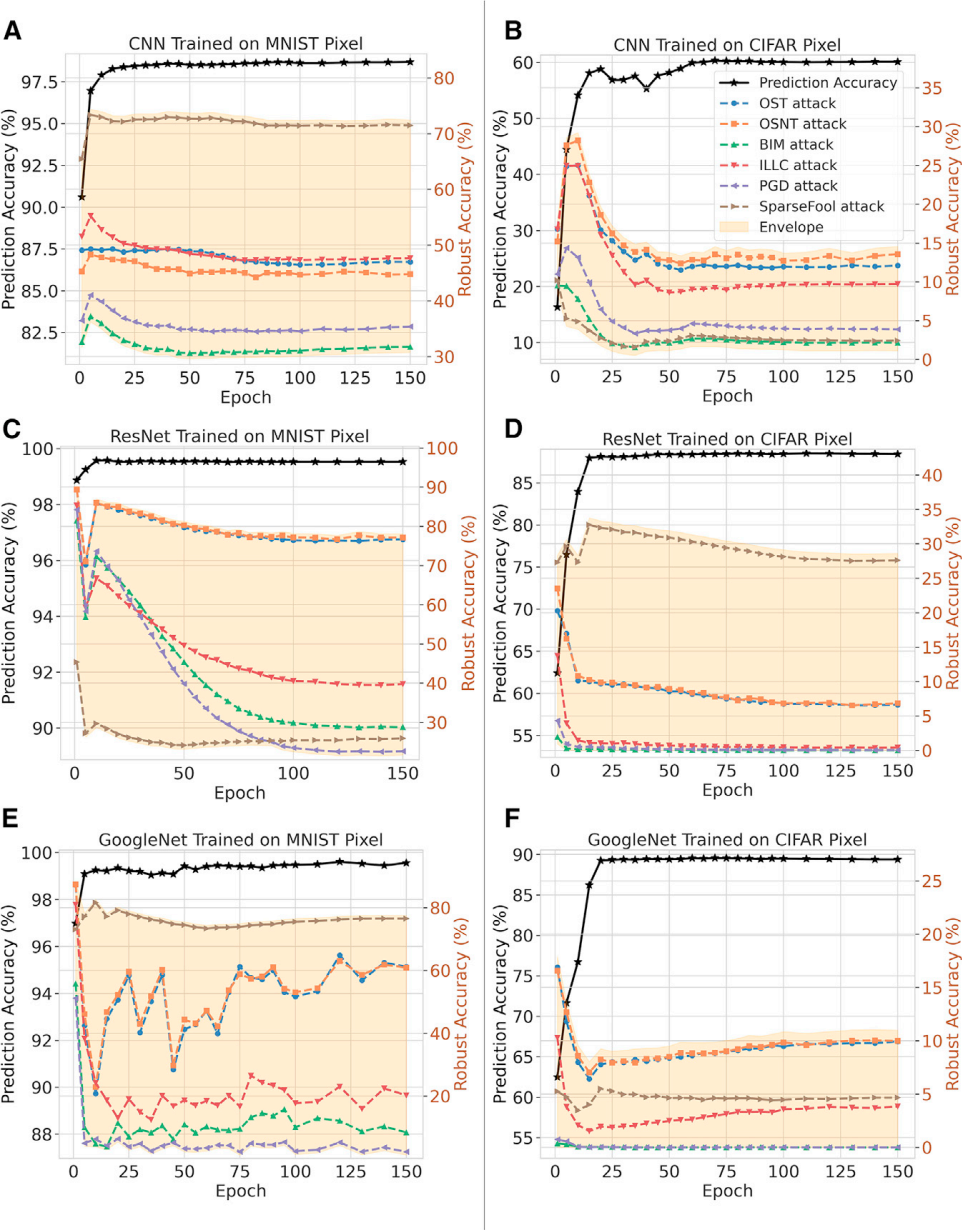
Test accuracy and robust accuracy under different attack methods for the MNIST and CIFAR-10 datasets The left panels (A, C, and E) are the results on MNIST dataset with CNN, ResNet, and GoogleNet; and the right panels (B, D, and F) give the corresponding results on CIFAR-10 dataset. In all subfigures, the black-dotted lines represent the test accuracies varying with the training epochs, while the other lines demonstrate the robust accuracies (i.e., the attacked accuracies). Networks are trained using a 4-layer CNN, ResNet, and GoogleNet. The black lines represent test accuracy, while the other six colored lines indicate robust accuracy. The attack amplitude for MNIST is 0.1, whereas for CIFAR-10, it is 8/255.

To see the trade-off between test and robust accuracies across various attack methods, we train three neural networks separately on the MNIST (an open source dataset containing handwritten digits from 0-9) and CIFAR-10 datasets: (1) a 4-layer CNN, (2) ResNet, and (3) GoogleNet. Here, robust accuracy refers to the accuracy when the network input is attacked. Due to the varying performances of different networks and the distinct pixel distributions of the datasets, the test accuracies of these networks versus epochs are shown by the black lines in Figure 2. For the MNIST dataset, all three neural networks achieve test accuracies over 98%. For the CIFAR-10 dataset, the simple CNN achieves only around 60% test accuracy, while ResNet and GoogleNet reach nearly 90%.

We then subject all networks to six types of gradient-based attacks: one-step target attack,^16^ one-step non-target attack,^16^ basic iterative method (BIM) attack,^29^ iterative least-likely class attack,^29^ projected gradient descent attack,^30^ and SparseFool attack.^31^ The other six lines in the figure represent the robust accuracies versus epochs after applying these attacks, and we draw a robust accuracy envelope to show the general variation trend. As observed, the general trend of test accuracy and robust accuracy is opposite: test accuracy increases with epochs, while robust accuracy under attack decreases simultaneously. Additionally, since the MNIST dataset is less complex, the robust accuracy only decreases by around 10%–15% after various attacks. However, for the CIFAR-10 dataset, the situation is more severe, with a decrease in robust accuracy of approximately 30%–50%. Furthermore, more complex neural networks trained on more intricate datasets are more susceptible to attacks.

### Phenomena in language model

To assess the vulnerability of large language models (LLMs) under small perturbation attacks, we conducted experiments in open-source LLM TinyLlama^32^ by introducing three types of perturbation into its embedding layer output: random attack, Fast Gradient Sign Method (FGSM), and BIM. Specifically, FGSM and BIM are gradient-based attacks, whereas random attack introduces gradient-independent random noise to the embedding vectors. All attacks were applied with a fixed amplitude of 0.01. The random and FGSM attack have the same amplitude and only differ in their directions. Subsequently, we evaluated the model’s performance using the Language Model Evaluation Harness^33^ on six multiple-choice benchmark datasets: ARC Challenge,^34^ ARC Easy, Hellaswag,^35^ OpenBookQA,^36^ PiQA,^37^ and Winogrande.^38^

**Figure 3:**
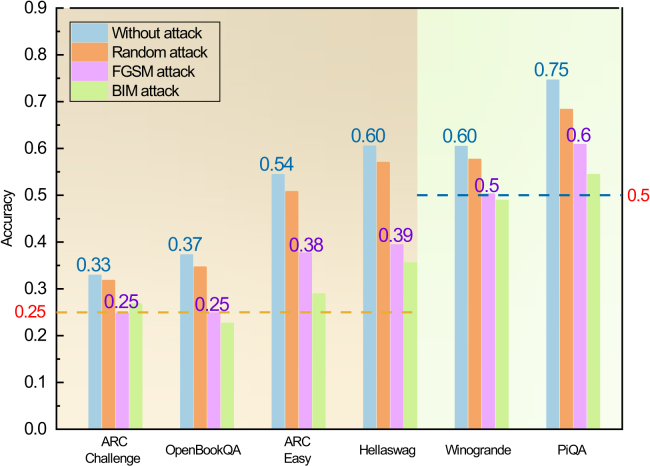
Performance of the TinyLlama model on six representative multiple-choice datasets under four distinct conditions The background is color-coded to distinguish dataset types: yellow for four-option datasets and green for two-option datasets. Dashed lines represent random guess accuracy (0.25 for four-option datasets and 0.5 for two-option datasets). Bars indicate the model’s evaluation accuracy, with annotations highlighting accuracy without attacks and the FGSM attacks.

The experimental results, as shown in Figure 3, provide insights into the model’s behavior under these attack scenarios. We see that all three types of attacks lead to a degradation in the performance of the model. However, gradient-based attacks cause significantly larger performance drops compared to random perturbations. In fact, for certain datasets, such as ARC Challenge, OpenBookQA, and Winograde, the model performance under gradient-based attacks decreases to the random guessing level. For instance, in the ARC Challenge dataset, where each question has four options, the model’s accuracy drops to 0.25 under both gradient-based attacks, indicating that it performs no better than random guessing. Similarly, for the Winograde dataset, where each question has two options, the model’s accuracy decreases to 0.5, again corresponding to the random guessing baseline.

## Discussion

### Neural networks exhibit the principle of complementarity similar to that found in quantum mechanics

We introduce an uncertainty principle for deep neural networks by drawing an analogy with the Heisenberg uncertainty principle in quantum mechanics. This principle describes an inherent trade-off between a neural network’s ability to recognize two conjugate variables: the input features and the corresponding gradients (gradients of the loss function with respect to these features). Based on the uncertainty principle, we demonstrate that the trade-off between the network’s prediction accuracy and its robustness to adversarial perturbations is a natural result of the uncertainty relation.

### Uncertainty principle paralleled from quantum mechanics for neural networks

We define a “neural packet” $\psi_{Y}\left(X\right)$ analogous to a quantum wave function, which is the normalized loss function of a network. By examining these diverse examples, we aim to highlight the widespread nature of neural network vulnerabilities, which contradicts the universal approximation theorem in response to input *X* and target *Y*,

(Equation 1) $$\psi_{Y}\left(X\right)=\frac{l\left(f\left(X,\theta \right),Y\right)}{\sqrt{\beta }}\text{,}$$

where the normalization coefficient *β* ensures that the integral of the squared neural packet over its input space equals one, $\int \psi_{Y}{\left(X\right)}^{2}dX=1$.

To obtain the uncertainty relation, we use the Dirac notation in quantum physics in the following contexts. For an input $X=\left(x_{1},\ldots ,x_{i},\ldots ,x_{M}\right)$ with *M* components in the multi-dimensional space, we introduce two operators, named the feature and attack operators, as follows,

(Equation 2) $$\begin{array}{c} {\hat{x}}_{i}\psi_{Y}\left(X\right)=x_{i}\psi_{Y}\left(X\right)\text{,}\\ {\hat{p}}_{i}\psi_{Y}\left(X\right)=\frac{\partial }{\partial x_{i}}\psi_{Y}\left(X\right)\text{.} \end{array}$$

Since $\psi_{Y}\left(X\right)$ is square normalized, its square can be seen as a “probability density,” allowing us to calculate the corresponding mean values of the two operators,

(Equation 3) $$\left\langle {\hat{x}}_{i}\right\rangle =\int \psi_{Y}\left(X\right)x_{i}\psi_{Y}\left(X\right))dX\text{.}$$

(Equation 4) $$\left\langle {\hat{p}}_{i}\right\rangle =\int \psi_{Y}\left(X\right)\frac{\partial }{\partial x_{i}}\psi_{Y}\left(X\right)dX\text{.}$$

It can be proved that the standard deviations $\sigma_{a}$ and $\sigma_{b}$ corresponding to two general operators $\hat{A}$ and $\hat{B}$ obey the following uncertainty relation (derivation is provided in the STAR Methods),

(Equation 5) $$\sigma_{a}\sigma_{b}={\left\langle {\left(\hat{A}-\left\langle \hat{A}\right\rangle \right)}^{2}\right\rangle }^{\frac{1}{2}}{\left\langle {\left(\hat{B}-\left\langle \hat{B}\right\rangle \right)}^{2}\right\rangle }^{\frac{1}{2}}\geq \left|i\frac{1}{2}\left\langle \left[\hat{A},\hat{B}\right]\right\rangle \right|\text{.}$$

Thus, in the context of neural networks, we can replace operators $\hat{A}$ and $\hat{B}$ by ${\hat{p}}_{i}$ and ${\hat{x}}_{i}$,

(Equation 6) $$\sigma_{p_{i}}\sigma_{x_{i}}\geq \left|i\frac{1}{2}\left\langle \left[{\hat{p}}_{i},{\hat{x}}_{i}\right]\right\rangle \right|=\frac{1}{2}\text{,}$$

where we have used the relation

$$\left[{\hat{p}}_{i},{\hat{x}}_{i}\right]\psi_{Y}\left(X\right)=\left[{\hat{p}}_{i}{\hat{x}}_{i}-{\hat{x}}_{i}{\hat{p}}_{i}\right]\psi_{Y}\left(X\right)$$

$$=\frac{\partial }{\partial x_{i}}\left[x_{i}\psi_{Y}\left(X\right)\right]$$

$$-x_{i}\frac{\partial }{\partial x_{i}}\psi_{Y}\left(X\right)$$

(Equation 7) $$=\psi_{Y}\left(X\right)\text{.}$$

The lower bound in Equation 14 arises from the normalization of the loss function, which is influenced by both the dataset and the network architecture. Consequently, for a trained neural network, the uncertainty relation is inherently dependent on the specific task (defined by the dataset) and the structure of the network. The detailed comparison of the formulas in quantum physics with those used in neural networks is tabulated in “(Table 1)” to facilitate easy understandings for interested readers.

**Table 1:** Comparison of the uncertainty principle between quantum physics and neural networks

Quantum physics		Neural networks	
Position	X=(x,y,z)	$X=\left(x_{1},\ldots ,x_{i},\ldots ,x_{M}\right)$	image (input)
Momentum (conjugate of position)	$P=\left(p_{x},p_{y},p_{z}\right)$	$P=\left(p_{1},\ldots ,p_{i},\ldots ,p_{M}\right)$	attack (conjugate of input)
Wave function	ψ(X)	$\psi_{Y}\left(X\right)$	normalized loss function (neural packet)
Normalize condition	$\int {\left|\psi \left(X\right)\right|}^{2}dX=1$	$\int {\left|\psi_{Y}\left(X\right)\right|}^{2}=1$	normalize condition
Position operator	${\hat{x}}_{i}\psi \left(X\right)=x_{i}\psi \left(X\right)$	${\hat{x}}_{i}\psi_{Y}\left(X\right)=x_{i}\psi_{Y}\left(X\right)$	pixel operator
Momentum operator	${\hat{p}}_{i}\psi \left(X\right)=-i\frac{\partial }{\partial x_{i}}\psi \left(X\right)$	${\hat{p}}_{i}\psi_{Y}\left(X\right)=\frac{\partial }{\partial x_{i}}\psi_{Y}\left(X\right)$	attack operator
Standard deviation for measuring position	$\sigma_{x_{i}}={\left\langle {\left({\hat{x}}_{i}-\left\langle {\hat{x}}_{i}\right\rangle \right)}^{2}\right\rangle }^{1/2}$	$\sigma_{x_{i}}={\left\langle {\left({\hat{x}}_{i}-\left\langle {\hat{x}}_{i}\right\rangle \right)}^{2}\right\rangle }^{1/2}$	standard deviation for resolving pixel
Standard deviation for measuring momentum	$\sigma_{p_{i}}={\left\langle {\left({\hat{p}}_{i}-\left\langle {\hat{p}}_{i}\right\rangle \right)}^{2}\right\rangle }^{1/2}$	$\sigma_{p_{i}}={\left\langle {\left({\hat{p}}_{i}-\left\langle {\hat{p}}_{i}\right\rangle \right)}^{2}\right\rangle }^{1/2}$	standard deviation for resolving attack
Uncertainty relation	$\sigma_{x_{i}}\sigma_{p_{i}}\geq \frac{1}{2}$	$\sigma_{x_{i}}\sigma_{p_{i}}\geq \frac{1}{2}$	uncertainty relation

### Explanation of the uncertainty principle of neural networks

#### Intuitive explanation

We use a binary example to explain the meaning of the quantities $\sigma_{x}$ and $\sigma_{p}$. A simple neural network is employed to classify data points based on their position relative to the origin. The dataset consists of points uniformly distributed in a 2D space, with labels assigned based on whether the points lie on the positive or negative side of the *x* axis. The neural network model comprises multiple fully connected layers and is trained over 500 epochs. Various metrics, including loss, accuracy, $\sigma_{x}=dx$, and $\sigma_{p}=dp$, are tracked throughout the training process. To ensure the reliability of our results, the experiment is repeated five times with different random seeds, and the average values are computed.

**Figure 4:**
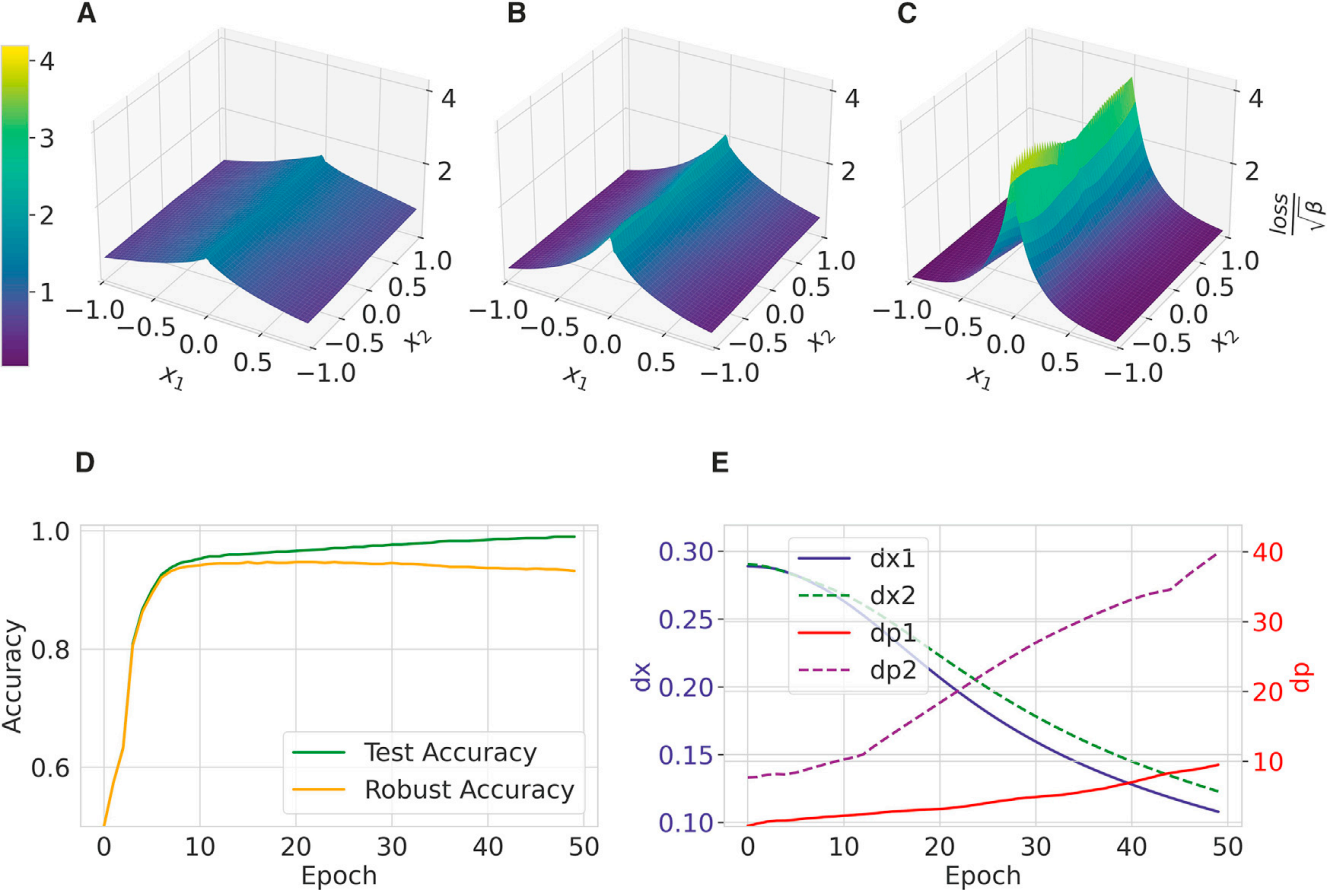
Explanation of the uncertainty relation via the binary classification (A–C) The loss landscapes at different training epochs 100, 200, and 500. The x and y axes represent the input features $x_{1}$ and $x_{2}$, respectively, while the z axis represents the normalized loss value $\psi \left(x1,x2,Label\right)=\frac{\text{loss}}{\sqrt{\beta }}$. These plots demonstrate how the loss landscape evolves during training, becoming sharper as the model converges. (D) The test accuracy (green) and robust accuracy under gradient attack (orange) as a function of training epochs. (E) The evolution of dx and dp values, which quantify the test accuracy and sharpness of the loss landscape. For convenience, here dx and dp are evaluated with $x_{2}=0$. Since the category interface between the two classes is similar along the $x_{2}$ direction, for convenience, we select a specific value for the loss function, i.e., ψ(x1,x2=0,Label). These plots highlight the trade-off between model accuracy and robustness, as well as the relationship between loss landscape sharpness and adversarial vulnerability. The results are averaged among 5 random seeds for reproducibility.

The results are presented in Figure 4. We observe that as test accuracy increases, robust accuracy gradually decreases. The boundary separating the two categories becomes steeper, leading to a decrease in dx. Concurrently, as dx decreases, the quantity dp increases, indicating the network’s vulnerability. This is because dp measures the sharpness of the boundary, which can also be understood from the Fourier transformation of the gradient:

(Equation 8) $$F\left\{\frac{\partial \psi_{Y}\left(x\right)}{\partial x}\right\}=\int_{-\infty }^{\infty }\frac{\partial \psi_{Y}\left(x\right)}{\partial x}e^{-ipx}\;dx=ip{\hat{\psi }}_{Y}\left(p\right)\text{.}$$

In real scenarios, the inputs often contain many concepts and features at different levels. Therefore, we encounter tremendous sharp boundaries that make the network vulnerable. dp, however, provides us with a quantitative measure of these many sharpnesses.

#### Quantum explanation

There are many concepts that are vital in quantum physics, such as wave packet, quantum state, uncertainty principle, quantum entanglement, state collapse, etc. Analogously, in the context of neural networks, even if it is not designed to mimic a quantum system, it can manifest some quantum aspects, such as the uncertainty principle and state collapse, to help us understand vulnerability phenomena and complementary effects.

We can treat the images that can be understood by human beings as eigenvalues of a neural network, as defined in Equation 2. In the surprisingly large space expanded by the network input, there are numerous images, but only those that mimic the real world and can be understood by human beings are the meaningful ones. In classification tasks, the classifier is fed with the eigenvalues (real images), and the network learns to mimic an operator where the normalized loss functions are the eigenstates (also called ontological states^39^), as defined in Equation 2. Similarly, for generative tasks, the neural network contains two procedures: to understand the inputs (similar to the classification task) and to produce correct outputs that can be understood by human beings. The latter can be seen as an artificial state collapse, hence a measurement. These analogies are essential for deriving the uncertainty relation; thus, we have defined and given meanings to these quantities in consistency with Equation 2.

The concept of a neural packet is quite similar to the concept of a wave packet. Since a neural packet is also square normalized to be unity, its square has the meaning of probability density. To see this, we again use the binary classification example. For images that have a higher probability of being recognized correctly, their squared loss values should be smaller. Those images near the boundaries are the most likely ones to be incorrectly categorized; hence, they have larger square values. It is worth noting that, contrary to the wave packet where larger square values indicate higher probability, the neural packet should be understood reversely in order to be consistent with the uncertainty relation.

The uncertainty relation is a genuine quantum phenomenon, which has a definite meaning in neural networks in terms of the complementarity principle. We can think of two aspects in understanding the images of the same network: one with loss landscape in the feature (input) space and one with loss landscape in the conjugate space. In both landscapes, accurate results should correspond to small dx or dp. Since accurate neural networks indicate smaller dx in feature space but result in larger dp in conjugate space, one cannot expect to obtain high accuracy in both feature and conjugate spaces. The attack method actually creates a scenario where information in feature and conjugate space is forced to be identified simultaneously (Equation 8), leading to a decline in accuracy.

However, it remains an open question whether one needs to treat neural networks as a non-linear version of a quantum system, since even in quantum mechanics there are still studies that consider quantum mechanics as a cellular automaton.^39^ Regardless of these arguments, it is beneficial, at least at the level of mathematics, for us to use the uncertainty relation in Equation 14 to perceive the complementary aspects of neural network vulnerability and provide different angles with conclusions that cannot be drawn by other theories. For instance, in training language models, if the training data contain complementary features, we may encounter abrupt collapse. In video generation, if the output is a complementary feature of the input, the correct physical laws cannot be learned by a single model. However, at the current stage, we still need more studies before we can conclude that neural networks are worth being mathematically modeled as quantum systems or that other quantum effects can be added to the network design for robustness.^40^

### Uncertainty principle essentially works at the feature level

It is natural to question the discrepancy between the observed phenomena in Figure 2 and the uncertainty relation derived in Equation 14, given that the pixels in the input images are dependent, while the uncertainty relation applies to an arbitrary dimension. In reality, the uncertainty relation operates at the level of features rather than individual pixels. As previously discussed, the uncertainty principle arises from the sharp boundaries between concepts and features, indicating that complementarity should manifest at the feature level and their corresponding conjugates. This is why we refer to the operator ${\hat{x}}_{i}$ as a feature operator rather than a pixel operator. Since the features of the input can be considered relatively independent variables, the uncertainty principle is more pronounced at the feature scale.

**Figure 5:**
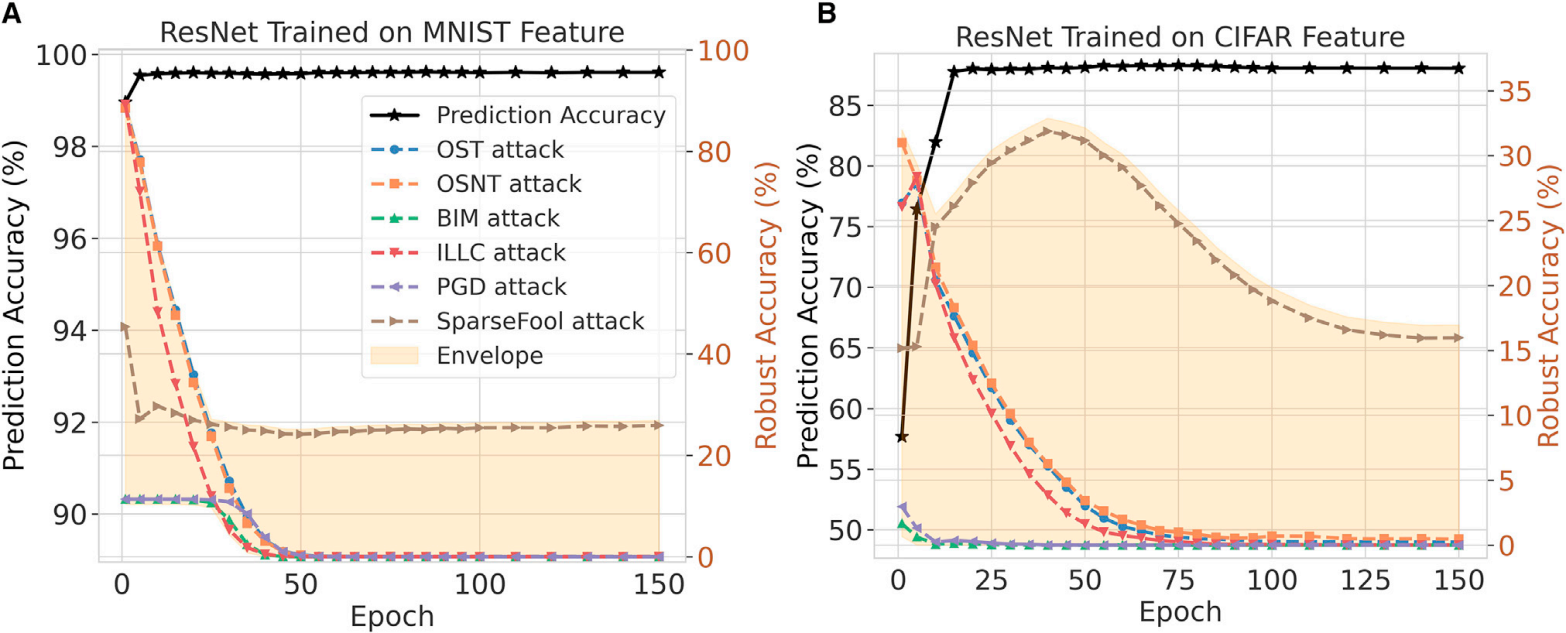
Test accuracy and robust accuracy under different attack on feature levels The left panel (A) gives the results on MNIST dataset with ResNet; and the right panel (B) gives the corresponding results on CIFAR-10 dataset. The ResNet for the MNIST and CIFAR-10 datasets in Figure 2 is divided into two parts: feature extractor and subsequent classifier. All attacks are performed on the outputs (hence, inputs of the subsequent classifier) of the feature extractor.

To illustrate this, we conducted experiments similar to those in Figure 2, but with the attack applied to the feature layers of the neural networks instead of the inputs. Because the specific features of the images cannot be exhaustively enumerated, we divided the neural network into two parts: the initial few layers, which extract features, and the subsequent layers, which perform classification. The attack was primarily executed on the outputs of the initial part (which also serve as the inputs to the latter part). The results are shown in Figure 5. Compared to Figure 2, the decline in robust accuracy with training epochs is more pronounced, supporting the underlying uncertainty relation.

### Role of complementarity in network optimization

The uncertainty principle of neural networks suggests that highly accurate networks tend to reach the lower bound of the uncertainty relation, which is influenced by the specific task and network structure. This principle can be understood through the complementarity principle,^24^^,^^41^ which addresses the mutually exclusive nature of two conjugate variables. In neural networks, these conjugate variables are the input and the gradient (gradient of the loss function with respect to the input). Consequently, we can anticipate that inputs correctly identified by the neural network should be as irrelevant as possible to the corresponding gradients, i.e., the neural network tends to avoid recognizing two conjugate variables simultaneously. Conversely, inputs that cannot be correctly identified must share more common features with the relevant gradients, so the network fails in recognizing these inputs correctly. This phenomenon should be more prominent in more accurate networks.

**Figure 6:**
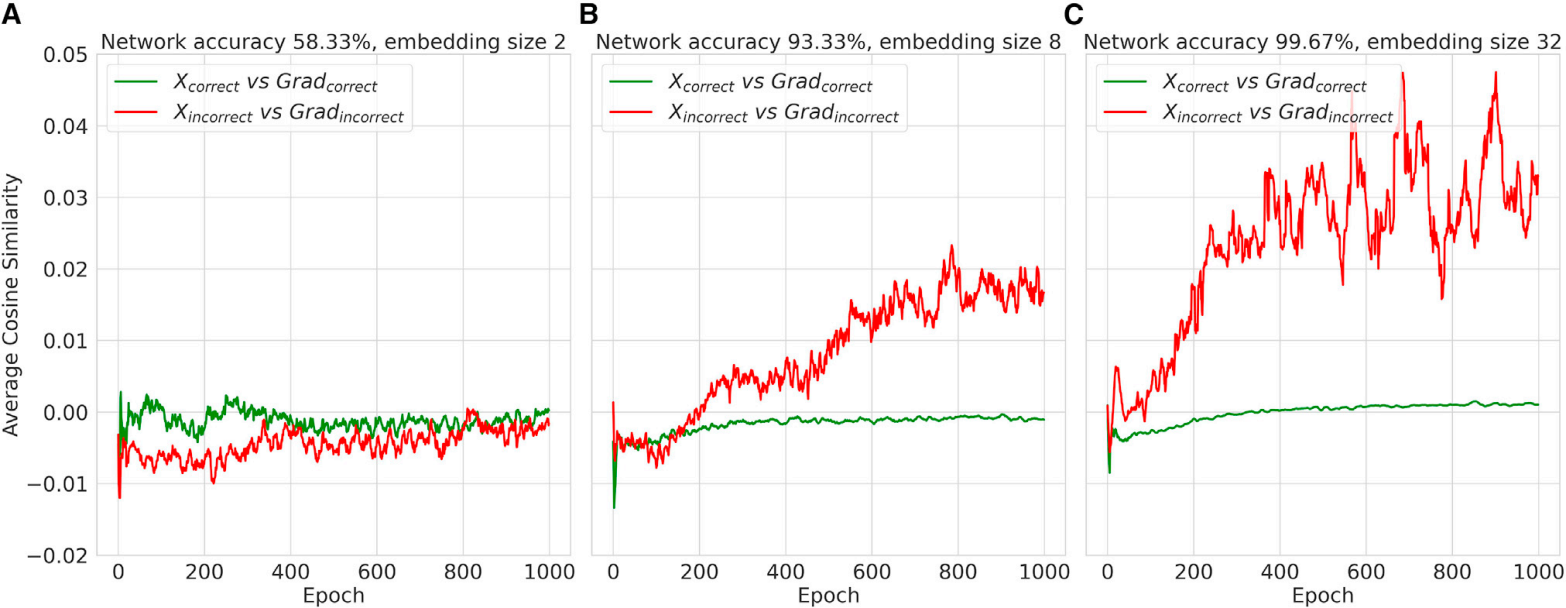
Cosine similarities between inputs and gradients Three types of words (positive, negative, and neutral) are fed to the neural network for sentiment classification. For each network model, similarities between correct input and the corresponding gradient, as well as the similarities between incorrect input and relevant gradients, are calculated at each epoch. The curves are averaged among 10 different random seeds for reproducibility. (A–C) give the cosine similarities with word embedding size of 2, 8, and 32, respectively. The differences in embedding size lead to different classification accuracies, i.e., 58.33%, 93.33%, and 99.67%, as labeled in the title of each subfigure.

To verify this hypothesis, we conducted an experiment involving word classification, categorizing words into positive, negative, and neutral sentiments. The dataset included a mix of English and Chinese words, which were labeled accordingly. These words were encoded and embedded into high-dimensional embedding vectors and were fed into a neural network model consisting of multiple fully connected layers for classification. The model was trained over 1,000 epochs with different embedding sizes to control the model accuracy. We then computed the cosine similarities between the input embeddings and their corresponding gradients for both correct and incorrect inputs, resulting in two types of similarities: correct input vs. correct gradient and incorrect input vs. incorrect gradient. The average cosine similarities for each category were tracked over the training epochs and collected for multiple random seeds to ensure reproducibility. The results, presented in Figure 6, confirm our anticipation: more accurate neural networks exhibit a more pronounced effect limited by the uncertainty relation, as manifested by the similarity between incorrect inputs and their corresponding gradients. Meanwhile, the similarity between correct inputs and the gradients tends to be zero for all models.

### Conclusion

Our work reveals that neural networks are subject to an uncertainty relation, similar to the uncertainty principle in quantum mechanics, through mathematical proofs and the concept of complementarity. We demonstrate that neural networks exhibit an inherent trade-off between accuracy and robustness, which can be quantified using the uncertainty relation. This trade-off explains why highly accurate neural networks tend to be more vulnerable to adversarial attacks.

Furthermore, our analysis shows that the vulnerability of neural networks is fundamentally due to the formation of sharp boundaries when learning different class concepts. Additionally, we use the complementarity principle to explain the difficulty in further optimizing highly accurate networks.

Our work suggests new directions for designing robust neural networks, such as exploring new architectures that can significantly separate concepts, or incorporating methods like Ohm’s global quantum potential^40^ to improve the network’s handling of sharp structures between concepts.

### Limitations of the study

While this study establishes the uncertainty principle as a fundamental factor underlying the accuracy-robustness trade-off in neural networks, several limitations warrant consideration. First, the uncertainty principle represents an inevitable constraint arising from the mathematical structure of neural networks, but it does not preclude other contributing factors to network vulnerability. For instance, architectural design choices (e.g., overparameterization, activation functions), optimization strategies (e.g., gradient descent dynamics), and dataset-specific biases may independently exacerbate or mitigate adversarial susceptibility. These factors could interact with the uncertainty principle in complex ways that remain to be fully disentangled.

Second, the practical quantification of the uncertainty bound remains challenging. The lower bound in Equation 14 depends intricately on the dataset distribution, network architecture, and training dynamics. Calculating this bound requires high-dimensional integration over the input space and loss landscape—a computationally intractable task for modern deep networks. Furthermore, the bound evolves during training as network parameters adapt, meaning the theoretical limit itself becomes a dynamic quantity. This complicates efforts to predict robustness thresholds for specific architectures or tasks.

Third, our analysis primarily addresses discriminative tasks (e.g., classification) where input-output mappings are sharply defined. The applicability of the uncertainty principle to generative models (e.g., diffusion models, generative adversarial networks) remains an open question. Such models operate through fundamentally different mechanisms—sampling from learned distributions rather than minimizing pointwise classification loss—whose vulnerabilities hide in the implicit features.

Lastly, while the complementarity principle provides a compelling analogy to quantum phenomena, the study does not establish whether neural networks inherently exhibit other quantum-like properties (e.g., entanglement, superposition) that might influence robustness. The theoretical framework focuses on conjugate feature pairs (input gradients vs. features), but real-world networks learn hierarchical representations with multi-scale correlations, potentially introducing higher-order uncertainty relations beyond the pairwise formulation explored here.

## Resource availability

### Lead contact

Requests for further information and resources should be directed to and will be fulfilled by the lead contact, Jun-Jie Zhang (zjacob@mail.ustc.edu.cn).

### Materials availability

This study did not generate new materials.

### Data and code availability

• The data have been deposited at https://doi.org/10.7910/DVN/FKFJZQ and are publicly available as of the date of publication. Accession numbers are listed in the key resources table.
• The Python code has been deposited at https://doi.org/10.7910/DVN/FKFJZQ and is publicly available as of the date of publication. Accession numbers are listed in the key resources table.
• Additional information required to reanalyze the data reported in this paper is available from the lead contact upon request.

## Acknowledgments

We thank Prof. David Donoho from Stanford University for providing valuable suggestions on the accuracy-robustness of neural networks.

The work was partly supported by the 10.13039/501100001809National Natural Science Foundation of China (NSFC) under grant number 62272375 and 12405318, the Major Key Project of PCL under grant PCL2024A06, and Tianyuan Fund for Mathematics of the 10.13039/501100001809National Natural Science Foundation of China under grant 12426105.

## Author contributions

Conceptualization, J.-J.Z. and D.M.; methodology, J.-J.Z., J.-N.C., L.-G.P., and D.-X.Z.; investigation, J.-J.Z., D.-X.Z., and J.-N.C.; writing – original draft, J.-J.Z., D.-X.Z., and J.-N.C.; writing – review and editing, L.-G.P. and D.M.; funding acquisition, D.M. and J.-N.C.; resources, D.-X.Z. and J.-J.Z.; supervision, D.M.

## Declaration of interests

The authors declare no competing interests.

## Declaration of generative AI and AI-assisted technologies

During the preparation of this work, the author(s) used Deepseek in order to polish the language. After using this tool or service, the author(s) reviewed and edited the content as needed and take(s) full responsibility for the content of the publication.

## STAR★Methods

### Key resources table

**Table:** 

REAGENT or RESOURCE	SOURCE	IDENTIFIER
**Deposited data**		

The work used the MNIST dataset. This dataset is sourced from THE MNIST DATABASE of handwritten digits. It's a subset of the larger NIST Hand-printed Forms and Characters Database published by National Institute of Standards and Technology.	Index of/exdb/mnist	MNIST
This work used the CIFAR-10 dataset. The CIFAR-10 datasets are labeled subsets of the 80 million tiny images dataset and is created by Alex Krizhevsky, Vinod Nair, and Geoffrey Hinton.	CIFAR-10 and CIFAR-100 datasets	CIFAR-10
This work used six multiple-choice benchmark datasets.	ARC Challenge/Easy can be found at https://huggingface.co/datasets/ai2_arc;Hellaswag can be found at https://huggingface.co/datasets/hellaswag; OpenBookQA can be found at https://huggingface.co/datasets/piqa, and Winogrande can be found at https://datasets-server.huggingface.co/parquet?dataset=winogrande	ARC Challenge34, 106 ARC Easy, Hellaswag, OpenBookQA, PiQA, and Winogrande.
Raw data used to shown the accuracy-robust trade-off.	https://doi.org/10.7910/DVN/FKFJZQ	Figures 2 and 5
Raw data used to shown the vulnerability of language model.	https://doi.org/10.7910/DVN/FKFJZQ	Figure 3
Raw data used to shown the uncertainty relation in binary classification.	https://doi.org/10.7910/DVN/FKFJZQ	Figure 4
Raw data used to shown the complementarity relation.	https://doi.org/10.7910/DVN/FKFJZQ	Figure 6

**Software and algorithms**		

Raw code in conducting the analysis.	https://doi.org/10.7910/DVN/FKFJZQ	Python code

### Method details

#### Experimental design

The study is designed to explore the trade-off between accuracy and robustness in neural networks, inspired by the uncertainty principle in quantum mechanics. The experiments are divided into three main categories: image classification tasks, language model tasks, and binary classification tasks. Each category evaluates the universality of the observed phenomena across different types of neural networks and tasks.

• Image Classification Tasks: We used widely adopted datasets, including MNIST and CIFAR-10, to evaluate the performance of neural networks under various attack scenarios. Three types of neural networks (4-layer CNN, ResNet, and GoogleNet) were trained to assess the universality of the observed phenomena across different architectures.
• Language Model Tasks: To assess the vulnerability of large language models (LLMs) under small perturbation attacks, we conducted experiments on the open-source LLM TinyLlama. We introduced three types of perturbation into its embedding layer output: Random attack, FGSM, and BIM. The model’s performance was evaluated on six multiple-choice benchmark datasets.
• Binary Classification Tasks: A binary classification task was designed to explain the uncertainty principle in a simplified setting. A neural network was trained to classify data points based on their position relative to the origin in a 2D space. The dataset consisted of points uniformly distributed in a 2D space, with labels assigned based on whether the points lie on the positive or negative side of the *x*-axis.

#### Data collection

The datasets used in this study were obtained from publicly available sources:

• MNIST: A grayscale image dataset of handwritten digits (60,000 training images and 10,000 test images).
• CIFAR-10: A color image dataset consisting of 60,000 32x32 images in 10 classes (50,000 training images and 10,000 test images).
• TinyLlama Datasets: Six representative multiple-choice datasets (ARC Challenge, ARC Easy, Hellaswag, OpenBookQA, PiQA, and Winogrande) were used to evaluate the language model.

The datasets were preprocessed to normalize pixel values to the range [0, 1] and augmentations such as random cropping and flipping were applied during training to improve generalization.

#### Attack methods

To evaluate the robustness of the models, we applied the following attack methods:

##### Image classification attacks

• FGSM (Fast Gradient Sign Method): A one-step attack that perturbs the input in the direction of the gradient of the loss function.
• PGD (Projected Gradient Descent): An iterative attack that refines the perturbation in the direction of the gradient within a bounded norm.
• BIM (Basic Iterative Method): A multi-step attack similar to PGD but with smaller step sizes.
• ILLC (Iterative Least-Likely Class Attack): An attack that iteratively minimizes the loss for the least likely class.
• SparseFool: A sparse attack that perturbs only a small fraction of the input features.
• Random noise: A non-adversarial attack that adds random Gaussian noise to the input.

##### Language model attacks

• Random attack: Introduces gradient-independent random noise to the embedding vectors.
• FGSM and BIM: Gradient-based attacks applied to the embedding layer output.

#### Uncertainty principle derivation

The uncertainty principle of a trained neural network can then be deduced by the following theorem: The standard deviations $\sigma_{p_{i}}$ and $\sigma_{x_{i}}$ corresponding to the attack and pixel operators $\hat{p_{i}}$ and $\hat{x_{i}}$, respectively, are restricted by the relation:

(Equation 9) $$\sigma_{p_{i}}\sigma_{x_{i}}\geq \frac{1}{2}\text{.}$$

Proof.

We first introduce the standard deviations $\sigma_{a}$ and $\sigma_{b}$ corresponding to two general operators $\hat{A}$ and $\hat{B}$. Then it follows that:

(Equation 10) $$\sigma_{a}\sigma_{b}={\left\langle {\left(\hat{A}-\left\langle \hat{A}\right\rangle \right)}^{2}\right\rangle }^{\frac{1}{2}}{\left\langle {\left(\hat{B}-\left\langle \hat{B}\right\rangle \right)}^{2}\right\rangle }^{\frac{1}{2}}\equiv {\left\langle {\hat{a}}^{2}\right\rangle }^{\frac{1}{2}}{\left\langle {\hat{b}}^{2}\right\rangle }^{\frac{1}{2}}\text{.}$$

In general, for any two unbounded real operators $\left\langle \hat{a}\right\rangle$ and $\left\langle \hat{b}\right\rangle$, the following relation holds

(Equation 11) $$0\leq \left\langle {\left(\hat{a}-i\hat{b}\right)}^{2}\right\rangle =\left\langle {\hat{a}}^{2}\right\rangle -i\left\langle \hat{a}\hat{b}-\hat{b}\hat{a}\right\rangle +\left\langle {\hat{b}}^{2}\right\rangle \text{.}$$

If we further replace $\hat{a}$ and $\hat{b}$ in Equation 11 by operators $\hat{a}{\left\langle {\hat{a}}^{2}\right\rangle }^{-1/2}$ and $\hat{b}{\left\langle {\hat{b}}^{2}\right\rangle }^{-1/2}$, we can then obtain the property 2⟨aˆ2⟩1/2⟨bˆ2⟩≥1/2i⟨aˆbˆ−bˆaˆ⟩, which gives the basic bound for the commutator $\left[\hat{a},\hat{b}\right]\equiv \hat{a}\hat{b}-\hat{b}\hat{a}$,

(Equation 12) $${\left\langle {\hat{a}}^{2}\right\rangle }^{\frac{1}{2}}{\left\langle {\hat{b}}^{2}\right\rangle }^{\frac{1}{2}}\geq \left|i\frac{1}{2}\left\langle \left[\hat{a},\hat{b}\right]\right\rangle \right|\text{.}$$

Seeing the fact that $\left[\hat{a},\hat{b}\right]=\left[\hat{A},\hat{B}\right]$, we finally obtain the uncertainty relation

(Equation 13) $$\sigma_{a}\sigma_{b}\geq \left|i\frac{1}{2}\left\langle \left[\hat{A},\hat{B}\right]\right\rangle \right|\text{.}$$

In terms of the neural networks, we can simply replace operators $\hat{A}$ and $\hat{B}$ by ${\hat{p}}_{i}$ and ${\hat{x}}_{i}$ introduced in Equation 2, and this leads to

(Equation 14) $$\sigma_{p_{i}}\sigma_{x_{i}}\geq \left|i\frac{1}{2}\left\langle \left[{\hat{p}}_{i},{\hat{x}}_{i}\right]\right\rangle \right|=\frac{1}{2}\text{,}$$

where we have used the relation

$$\left[{\hat{p}}_{i},{\hat{x}}_{i}\right]\psi_{Y}\left(X\right)=\left[{\hat{p}}_{i}{\hat{x}}_{i}-{\hat{x}}_{i}{\hat{p}}_{i}\right]\psi_{Y}\left(X\right)$$

$$=\frac{\partial }{\partial x_{i}}\left[x_{i}\psi_{Y}\left(X\right)\right]$$

$$-x_{i}\frac{\partial }{\partial x_{i}}\psi_{Y}\left(X\right)$$

(Equation 15) $$=\psi_{Y}\left(X\right)\text{.}$$

Note that for a trained neural network, $\psi_{Y}\left(X\right)$ depends on the dataset and the structure of the network.

Equation 14 is a general result for general neural networks (see extension to the generation network in supplementary material). For convenience, we compare the formulas in quantum physics with those used in neural networks in “(Table 1)” to facilitate easy understandings for readers.

In the FGSM attack, the attacked image is of the form:

$$X=X_{0}+\epsilon \cdot \text{sign}\left(\nabla_{X}l\left(f\left(X,\theta \right),Y^{*}\right){|}_{X=X_{0}}\right)$$

$$∼X_{0}+\epsilon \cdot \nabla_{X}l\left(f\left(X,\theta \right),Y^{*}\right){|}_{X=X_{0}}$$

$$=X_{0}+\epsilon \cdot \nabla_{X}\left[\beta^{1/2}\psi_{Y^{*}}\left(X_{0}\right)\right]$$

(Equation 16) $$=X_{0}+\epsilon^{\prime }\hat{P}\psi_{Y^{*}}\left(X_{0}\right)\text{,}$$

where $\hat{P}=\left(\frac{\partial }{\partial x_{1}},\ldots ,\frac{\partial }{\partial x_{i}},\ldots ,\frac{\partial }{\partial x_{M}}\right)$ and $\epsilon^{\prime }=\epsilon \cdot \beta^{1/2}$. In the second line of Equation 16 we have used the property substantiated in^42^: “even without the ‘Sign’ of the FGSM, a successful attack can also be achieved”. From Equation 16, we can then obtain

(Equation 17) $$\hat{P}\psi_{Y^{*}}\left(X_{0}\right)∼\epsilon /\epsilon^{\prime }\cdot \text{sign}\left(\nabla_{X}l\left(f\left(X,\theta \right),Y^{*}\right){|}_{X=X_{0}}\right)\text{,}$$

which is the reason that we call $\hat{p_{i}}$ the attack operator.

#### Feature-level attack analysis

To investigate the uncertainty principle at the feature level, we modified the attack to target the feature representations of the neural network instead of the raw input pixels. The network was divided into two parts: a feature extractor and a classifier. Attacks were applied to the outputs of the feature extractor, and the robust accuracy was evaluated under these feature-level attacks.

#### Complementarity principle analysis

To verify the complementarity principle, we conducted experiments on sentiment classification tasks. We computed the cosine similarity between input embeddings and their corresponding gradients for both correct and incorrect inputs. The results were averaged across 10 random seeds to ensure reproducibility.

### Quantification and statistical analysis

#### Metrics

The following metrics were used to evaluate the performance of the models:

• Test accuracy: The proportion of correctly classified samples in the test set.
• Robust accuracy: The proportion of correctly classified samples under adversarial attacks.
• Sharpness of the loss landscape: Quantified using the standard deviation of the gradient ($\sigma_{p}$) and input features ($\sigma_{x}$).

#### Software and tools

The following software and tools were used in this study:

• Python: The primary programming language.
• PyTorch: Used for implementing and training the neural networks.
• FGSM, PGD, and other attack implementations were adapted from the CleverHans library.

### Additional resources

• Code for reproducing the experiments: https://doi.org/10.7910/DVN/FKFJZQ.
• Data for the experiments: https://doi.org/10.7910/DVN/FKFJZQ.
• Supplementary materials, including extended derivations and additional experiments, are available upon request from the lead contact.
